# Combining Multiple Algorithms for Road Network Tracking from Multiple Source Remotely Sensed Imagery: a Practical System and Performance Evaluation

**DOI:** 10.3390/s90201237

**Published:** 2009-02-24

**Authors:** Xiangguo Lin, Zhengjun Liu, Jixian Zhang, Jing Shen

**Affiliations:** 1 Key Laboratory of Mapping from Space of State Bureau of Surveying and Mapping, Chinese Academy of Surveying and Mapping, 16, Beitaiping Road, Haidian District, Beijing 100039, P.R. China; E-Mails: linxiangguo@gmail.com (X.L.); zhangjx@casm.ac.cn (J.Z.); 2 School of Resources and Environment, Wuhan University, 129, Luoyu Road, Wuchang District, Wuhan 430079, P.R. China; E-Mail: shenjing00@163.com (J.S.)

**Keywords:** Semi-automatic, road tracking, profile matching, template matching, angular texture signature, parallelepiped classification, lane marking

## Abstract

In light of the increasing availability of commercial high-resolution imaging sensors, automatic interpretation tools are needed to extract road features. Currently, many approaches for road extraction are available, but it is acknowledged that there is no single method that would be successful in extracting all types of roads from any remotely sensed imagery. In this paper, a novel classification of roads is proposed, based on both the roads' geometrical, radiometric properties and the characteristics of the sensors. Subsequently, a general road tracking framework is proposed, and one or more suitable road trackers are designed or combined for each type of roads. Extensive experiments are performed to extract roads from aerial/satellite imagery, and the results show that a combination strategy can automatically extract more than 60% of the total roads from very high resolution imagery such as QuickBird and DMC images, with a time-saving of approximately 20%, and acceptable spatial accuracy. It is proven that a combination of multiple algorithms is more reliable, more efficient and more robust for extracting road networks from multiple-source remotely sensed imagery than the individual algorithms.

## 1. Introduction

The increasing availability of commercial high-resolution satellite imaging sensors such as SPOT5, IKONOS, QuickBird and TerraSAR, requires the availability of suitable automatic interpretation tools to extract and identify cartographic features, especially in rapidly changing urban areas. Roads are one of the most important linear cartographic features. Particularly, extraction of road networks from remotely sensed imagery is not only meaningful for cartography and topography [1], but also significant for various applications of geodata such as automatically aligning two spatial datasets [2] or automated vehicle navigation [3]. Therefore, research on the automatic extraction of road networks from remotely sensed imagery has been a topical research theme in the various fields of photogrammetry, remote sensing, geographic information systems, pattern recognition, and computer vision. As a result, many strategies, methodologies and algorithms for road network extraction have been presented since the 1970s, which have achieved varying degrees of success [4]. According to the level of automation, the techniques for road extraction with the aid of computer vision can be coarsely classified into automatic and semi-automatic approaches.

The automatic methods attempt to seek an analysis and interpretation of the image similar to that of a human operator. Nevatia and Babu [5] utilized an edge detection method to identify ribbon roads with lateral and parallel anti-edges. Radon transform was employed to locate the roadsides and to measure the width of a road [6]. Due to the variation of the complexity of image contents, the above low level edge detection methods are insufficient to extract the road features with high completeness, correctness, quality and accuracy. Therefore, more high level techniques have been developed. For example, angular texture signatures can make use of the characteristics of road's texture, and this is utilized to find candidate road centerline points [7] or to discriminate road surfaces from the parking lots [8]. Mathematical morphology is not only useful to dilate and enhance the road surfaces and roadsides [9], but also helpful to extract the skeletons of ribbon roads [10]. Segmentation and classification methods are popular due to the fact that they could utilize road's radiometric, geometrical, topological, and elevation characteristics to help finding road networks [11,12]. Heipke *et al.* [13] utilized a multi-scale strategy to extract global road network structures initially at a low resolution and detailed substructures later at a high resolution. Multi-view approaches not only can reconstruct 3D models, but also can utilize the multi-cues from multiple source images [14,15]. Rule-based approaches use reasoning methods to deal with the problems of segment alignment and fragmentation, as well as enable bottoms-up processing to link the fragmented primitives into a road network [16]. Statistical inference methods were also used to model the road linking process as a geometric-stochastic model [17], an active testing model [18], a MRF-based model [19], or a Gibbs point process [20]. Another category of automatic approaches is the use of existing information or knowledge to guide road extraction [21]. Currently, the tendency is that more and more methodologies are based upon hybrid strategies. For example, profile analysis, rule-based linking and model-based verification are combined together to detect, trace and link the road segments to form a road network [22]; Hu *et al.* [2] combined a spoke wheel operator, used to detect road surfaces, and a toe-finding algorithm, utilized to determine the road direction, to trace roads; multi-resolution and object-oriented fuzzy analysis is integrated to extract cartographic features [23]; and a novel combination strategy was adopted by Peng *et al.* [24] who incorporated an outdated GIS digital map, multi-scale analysis, a phase field model and a higher order active contour to extract roads from very high resolution (VHR) images. Despite the fact that much work on automatic approaches for road extraction has taken place, the desired high level of automation could not be achieved yet [25]. The main problem of a fully automatic approach is that it needs some strict hypothesis of road characteristics, but road properties vary considerably with ground sampling distances (GSD), road types, and densities of surrounding objects, light conditions etc. Therefore, the quality of automatic extraction is usually insufficient for practical applications.

On the other hand, semi-automatic methodologies are considered to be a good compromise between the fast computing speed of a computer and the efficient interpretation skills of a human operator [1], and quite a number of promising approaches for semi-automatic road extraction have been proposed so far. Optimal search methods, which are often realized by dynamic programming [26] or snakes [27], are frequently applied to find or determine an optimal trajectory between manually selected seed points. In these models, geometric and radiometric characteristics of roads are integrated by a cost function or an ‘energy’ function. Then the road extraction is equivalent to seeking the global energy minimum. However, it is hard to define the reasonable ‘energy’ function for each image. Angular texture signature (ATS) has also been improved to semi-automatically track road axes. Lin *et al.* [28] and Shen *et al.* [29] proposed the mean value and the entropy besides existing variance to measure the texture. As a result, ATS is capable of tracing the bright ribbon roads from SPOT imagery or tracing the dark ribbon roads from VHR COSMO SAR imagery. Minimum cost to follow a path was proposed by Shukla *et al.* [30]. Essentially, the minimum cost is similar to the ATS in the sense that it takes variance as a measure of texture. Another practical methodology is template matching. McKeown and Denlinger [31] introduced a semi-automatic road tracker based on profile correlations. This road tracker starts from some road seed points. The profile matching technique compares a reference profile with the road profile at a pixel predicted to be on the road. The differences between the two profiles are measured by identifying two geometric parameters (shift and width) and two radiometric parameters (brightness and contrast). These parameters are estimated by minimizing the squared sum of the gray value differences between the profiles. Actually, the subsequent approaches essentially follow the same scheme as the above method. Vosselman and Knecht [1] improved the profile matching with a Kalman filter, Baumgartner *et al.* [25] also developed a human-computer interactive prototype system based on the above method. Zhou *et al.* [32] used two profiles, one orthogonal to the road direction and the other parallel to the road direction, to enhance the robustness of the tracker and applied extended Kalman filter and particle filter to solve profile matching problems for road tracking, and a prototype system for semi-automatic road extraction is also developed. Kim *et al.* [33] utilized a rectangular template instead of profile to track ribbon roads by least squares template matching from VHR satellite images. Zhao *et al.* [34] used rectangular template matching on the classified imagery and proposed another prototype. However, there are no reports on their applicability on VHR images finer than 0.2 m/pixel, and most of the road trackers mentioned above don't operate well when they encounter irregular geometric deformations and radiometric changes due to the appearance of road junctions, material changes, occlusion from cars, shadows, lane markings etc. As a result, consecutive failure of the road tracker not only loses efficiency, but also increases the work required of the human operators.

After reviewing the existing work on road extraction, it was realized that ATS, profile matching and rectangular template matching are practical to extract road networks. However, it is acknowledged that there is no single method which is successful in extracting all types of roads from any remotely sensed imagery. Moreover, it is believed that a number of techniques developed for different classes of roads and for different kinds of imagery will lead to a many-branched solutions for road extraction which will be effective for a wide range of road types. In this paper, roads are classified into the ones with lane markings and the ones without lane markings on VHR remotely sensed imagery whose GSDs range from 2.5 per pixel to 0.2 m per pixel. Furthermore, two road trackers are proposed to extract the roads. Particularly, the profiles and rectangular templates are combined together to form an interlaced template, while the ATS [28,29] is further improved by a parallelepiped classifier, named parallelepiped angular texture signature (PATS). Correspondingly, a general road tracking framework is proposed. Then least squares interlaced template matching is applied to extract ribbon road networks with salient road markings, while the profile matching, rectangular template matching, and PATS are combined to extract ribbon roads without salient lane markings. In general, a practical system, combining multiple road trackers, is designed to extract the roads from VHR images in this paper.

The remaining sections of this paper are organized as follows. In Section 2, a general scheme of road tracking is introduced, and the interlaced template matching and the PATS are proposed. In Section 3, experiments are described and the performances of the road trackers are evaluated and discussed. In Section 4, some conclusions are reached.

## 2. Methods

### 2.1. The general framework

**Figure 1.**
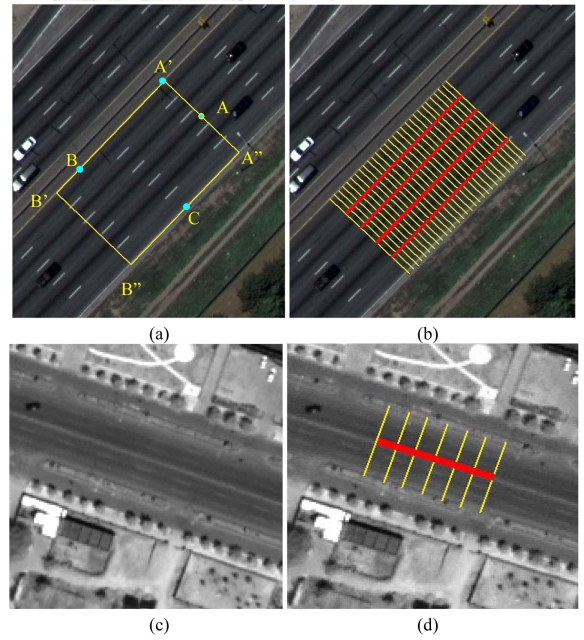

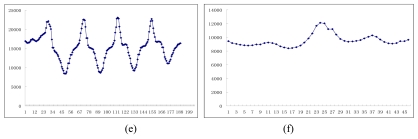
Construction of the interlaced template and detection of the road markings. (a) A subset of color aerial image and the initialization of our road tracker; (b) the interlaced template of the road surface in (a); (c) a subset of a panchromatic satellite image; (d) the interlaced template of the road surface in (c); (e) the profile transformations of the ribbon road in (b) and there are four salient peaks representing the four lane markings on the road surface; (f) the profile transformations of the ribbon road in (d) and there is only one salient peak representing a marking line on the road surface.

In the semi-automatic plotting system, a human operator is required in the annotation process where computer algorithms are utilized to assist the plotter performing measurement tasks. From the user's point of view, the procedure is as follows: the operator first identifies a short segment of a road which serves as initialization for an automatic tracking, and then the tracker algorithms are launched and automatically trace the road axis for as long as possible. Whenever the internal evaluation of the algorithms indicates that the tracker might have lost the road centerline, it needs intervention of the user. Then the operator has to confirm the tracker or he/she must edit the extracted road and put the tracker back the road again. Particularly, the semi-automatic tracking is divided into the following steps:

1) Initialization by three seed points. Similar to McKeown's strategy [31], in this paper the initialization is also accomplished by manual selected seed points. However, we make an improvement in that a three consecutive mouse clicks strategy is adopted to obtain the starting location, direction, width of the road, and the step size as well. This three seeds method is feasible for most ribbon roads, and it is accomplished as follows [see Figure 1(a)]: the human operator enters a road segment with two consecutive mouse clicks on A′ and B with the axis joining the points defining one road sideline A′B, which indicates road direction arctangent(A′B), then the following third click on C, on the other roadside, defines the width *w* of the road. *w* is equal to the distance between the point C and the line A′B. As a result, the above three points can also derive a rectangle A′B′B″A″ with width *w* and length *L_sign_*. And the direction of A′B′ is equal to arctangent(A′B) while C is located on the side B″A″. Then a start point A of the road is derived from the middle point of A′ A″. In this paper, *L_sign_* is equal to a coefficient, *C_sign_*, multiplying by the road width *w*.

Subsequently, the operators can select any road tracker from profile matching, template matching, interlaced template matching (introduced in Section 2.2), PATS (introduced in Section 2.3) based on their judgments. Alternatively, the computer can automatically adapt a combination strategy based on the following procedures. First, it is necessary to detect whether there is lane marking in the rectangle A′B′B″A″. Correspondingly, a new method is proposed to automatically determine the number of lane markings *N_sign_* and the road markings' positions along the road profile to enhance the automation. In fact, a strip of lane marking is a brighter bar-shaped ribbon [14]. Suppose there is a profile *P_profile_*, with length *w* and direction arctangent $({\text{A}}^{\prime }\text{B})+\frac{\pi }{2}$, and it passes the start point A. Then *P_profile_* derives a series of profiles with length *L_sign_* and direction arctangent(A′B) at each pixel of *P_profile_*. At last, we calculate the sum of the derived intensity profiles at each pixel of *P_profile_* one by one, which will form a line chart. Therefore, a significant peak of this line chart corresponds to a brighter marking in the image, and the above process is named a profile transformation (PT) in this paper. For example, two PTs are shown in Figures 1 (e) and (f), respectively. It is clear that the first PT has four dominating peaks while the second PT has only one dominating peak. However, there are some weak peaks or small peaks on or near dominating peaks [see Figures 1 (e) and (f)], which make precisely locating the dominating peaks difficult. The toe-finding algorithm [2] is employed to find the number of dominant peaks that represent road directions and simultaneously locate road markings' positions on *P_profile_*. This toe-finding approach is divided into two separate steps. First, the dominant peaks are found. Second, valleys between sequential peaks are found. In the algorithm, there are three constant coefficients, ∈_1_, ∈_2_, and ∈_3_, and they are selected based on our experiments. We set ∈_1_ = 0.25, ∈_2_ = 0.8, and ∈_3_ = *w*/8, i.e., if the distance of two peaks is less than *w*/8, then merge the two peaks.

As long as any lane marking found, generate a reference interlaced template described in Section 2.2, and then interlaced template matching is triggered. If there are no markings, the standard deviation *σ* of intensity rectangle A′B′B″A″ is calculated. In this circumstance:

- If *σ*<*I*_1_ hold, generate a reference profile with width *W_profile_*, and go to the profile matching algorithm;
- If *σ*>*I*_1_ and *σ*<*I*_2_ hold, generate a reference rectangular template with width *w* and length *L_sign_*, and go to the template matching algorithm;
- If *σ*>*I*_2_ hold, go to the PATS algorithm described in Section 2.3.

2) Acquire the next road axis point. If the interlaced template matching, profile matching or template matching is launched, the system then predicts next most possible position of the road axis according to the following equation:

(1) $$\left[\begin{array}{c} x_{\text{next}}\\ y_{\text{next}} \end{array}\right]=\left[\begin{array}{cc} \cos\theta_{\text{rotating}} & -\sin\theta_{\text{rotating}}\\ \sin\theta_{\text{rotating}} & \cos\theta_{\text{rotating}} \end{array}\right]*\left[\begin{array}{c} x_{\text{current}}+L_{\text{step}}*\cos\theta_{\text{current}}+S*\cos(\theta_{\text{current}}+\frac{\pi }{2})\\ y_{\text{current}}+L_{\text{step}}*\sin\theta_{\text{current}}+S*\sin(\theta_{\text{current}}+\frac{\pi }{2}) \end{array}\right]$$

where *x_next_, y_next_* are the coordinates of next template point; *x_current_, y_current_* are the coordinates of the current template point; *L_step_* is the suitable step size of the increment (i.e., the distance between two consecutive points on the road axis); *S* is a variable indicating the shift distance around the predicted point in convolution; *θ_current_* is the direction of the current road axis point; and *θ_rotating_* is a variable indicating the change/offset in road direction. In this paper, *L_step_* is equal to a coefficient, *C_steplength_*, multiplying by the road width *w*, and *θ_rotating_* ∈ (0°, ±5°, ± *T*°), *S* ∈ (0,±1, ±2,…, ± (*w*/8)).

When the reference template is convolved within part of the image by Equation (1), a number of target templates are generated. The one which has the minimal squared sum of gray value differences between reference template and target template is taken as the optimal response. With this optimal response, the next road axis is located.

Alternatively, if PATS is triggered, the operation is performed as follows: revolve the current road axis point *p* and calculate the PATS values; take the direction of the significant maximum which has a minimal inclination with the current road direction *θ_current_* as the direction of next road axis point; and get the next road axis point by Equation (1) where *θ_rotating_*=0°, *S*=0.

3) Validate the above optimal point. Once the above obtained point is added into the road trajectory, check whether any stopping criterion is fulfilled as follows:

- the change of the directions of two adjacent road segments is larger than predefined threshold *T*;
- approaching an extracted road or border of the image;
- the minimal squared sum of gray value differences between the reference template and the target template surpass *T*_1_ for the interlaced template matching, profile matching or template matching;
- compactness of PATS polygon [8] is larger than *T*_2_.

If any of these conditions is encountered, exit the tracking procedure and go to Step 4). Otherwise, go to Step 2) again.

4) Stop the trace. If no rule can be made to continue the tracking procedure, the system will stop tracking, report the reason, and offer an appropriate choice of user interaction. The user can then modify the traced path with the aid of common GIS-functionalities, manually digitize complex roads, update the reference template (occurrence of change of the number of lanes, or significant change of spectral characteristics due to different ages, construction materials, illumination angles, etc.), or restart the tracking process from the next specified location.

In this paper, for each test image, the four state-of-the-art road algorithms, such as the profile matching, rectangular template matching, interlaced template matching, PATS, and their combination strategy were employed, respectively, to trace the road network to compare their performances. In the tracking procedure, for each type of road, if a road tracker frequently fails, it suggests the road tracker is not suitable for tracing this type of road, and then another type of road is tried. Moreover, manual digitization was adopted to create reference data to verify the performances of the road trackers. Note that in the process of full manual annotation, the manual clicks should be as accurate as possible, i.e., the selected points should be on the road axes. Furthermore, the digitized roads should be smooth, i.e., abrupt changes in directions should be avoided and no zigzags should occur.

### 2.2. The interlaced template matching

In fact, the salient lane markings are usually less frequently disturbed by the occlusions of cars or the shadows of colonnade than other parts of road surfaces [35] on VHR imagery. This meaningful phenomenon is neglected by most of existing research about semi-automatic road tracking. In this case, if templates of lane markings rather than the template of a segment of whole rectangular road surface are utilized in template matching, it may be more effective and robust. Considering that the above salient road markings are often discontinuous, it is a tradeoff between a rectangular template of road surface and some templates of salient lane markings. Consequently, we can take a novel template, i.e., an interlaced template which is composed of two parts: some cross-section profiles (i.e., each is a typical intensity profile perpendicular and symmetrical to the road axis) and some rectangular templates of road markings (i.e., some intensity rectangles whose width are as wide as lane markings).

Normally, the intensity of pure road surface except the disturbing features tends to be constant, and so are the intensities of road markings. This characteristic can be formulated as follows:

(2) $$\sum_{i}^{N_{\text{profile}}}\sum_{j}^{W_{\text{profile}}}{\left(g-g\prime \right)}^{2}+\sum_{k}^{N_{\text{signs}}}\sum_{l}^{L_{\text{sign}}}\sum_{n}^{W_{\text{sign}}}{\left(m-m\prime \right)}^{2}\to \min\;(\min<T_{1})$$

where *g, g′* are the gray values of two homonymy points on the profiles, *m, m′* are the gray values of two homonymy points on the rectangular templates of lane markings, *N_profile_* is the number of profiles in the interlaced template, *W_profile_* is the length of the profile, *N_sign_* is the number of markings, *L_sign_* is the length of the markings, *W_sign_* is the width of the road marking that is a preset value inputted by the operator based on the GSD, and *T*_1_ is an adaptive value. Actually, Equation (2) is a specific expression of least square method [1] for interlaced template matching. In this paper, *W_profile_* is equal to a coefficient, *C_profile_*, multiplying by the road width *w*; and the number of profiles *N_profile_* is derived by the following formula:

(3) $$N_{\text{profile}}=(N_{\text{signs}}*L_{\text{sign}}*W_{\text{sign}})/W_{\text{profile}}$$

Equation (3) guarantees that the number of pixels in the profiles is equal to the one of pixels of lane markings, and the equality has significant effect on determining the threshold *T*_1_. In this paper, *T*_1_ is equal to variance of the profiles in the reference interlaced template plus variance of the markings in the reference interlaced template. In this sense, *T*_1_ is adaptive in our paper.

Once *N_sign_* and road markings' positions are decided by PT and toe-finding algorithm, the rectangular templates of markings are constructed. Then *N_profile_* can be deduced by Equation (3), and the profiles can be constructed. The interval between two profile is max (*L_sign_*/(*N_profile_*-1),1). Note that if *L_sign_* < (*N_profile_*-1), then the profiles evolve into the rectangle template [33]. Figure 1 (b) and (d) illustrate two examples of interlaced templates respectively.

### 2.3. PATS

**Figure 2.**
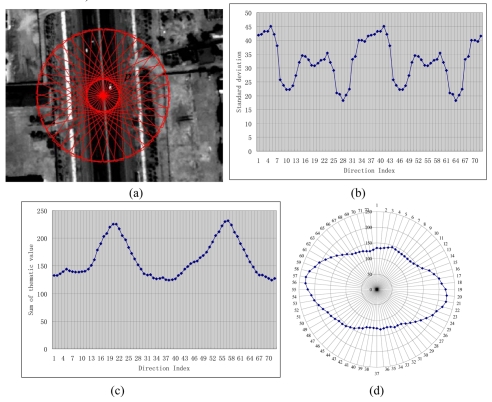
Angular Texture Signature. (a) Texture is computed over a set of rectangular regions rotating around a road centerline point (note that there are 72 templates but only odd ones are displayed); (b) the graph of the ATS; (c) the graph of the PATS; (d) the PATS polygon of (c).

A texture measure is described in [7]. Practically, it is hard to populate ATS if it still takes variance as a measure of texture in complex scene where even intensities of one feature differ greatly. Fortunately, it could still function on a classified thematic image. As mentioned above, the initialization information derives a rectangle A′B′B″A″. And then for each band, calculate the mean M and standard deviation *σ* of the gray values of pixels in rectangle A′B′B″A″ respectively, then perform parallelepiped classification using ± *σ* as limits on the raw image. Subsequently, set the pixels of road subclass as 1, meanwhile set the pixels of any other subclass as 0, and then the mean of the rotating template is feasible too [see Figure 2(c)].

In this sense, PATS is improved and redefined to make it fit to semi-automatic road tracking as follows. At each road centerline point *p, T*(*α, w, h, p*) is defined as the mean for a rectangular set of pixels of width *w* and height *L_sign_* around pixel *p* whose principal axis lies at an angle of *α* from the road direction *β*. This measure is computed for a set of angles *α*_0_, …, *α_n_* at pixel *p*. Angles *α*_0_, …, *α_n_* are with same interval *δ*. At the point *p*, the ATS is regarded as the set of values {*T*(*α*_0_, *w*, *h, p*), *T*(*α*_1_, *w, h, p*), …, *T*(*α_n_, w, h, p*)}. Figure 2(a) displays an ATS with *δ*=5°, Figure 1(c) shows the improved PATS values related to Figure 2(a). The direction of the significant maximum which has a minimal inclination with road direction *β* is taken as the direction of next road axis point.

To find the relationship between the shape of the PATS polygon and corresponding pixel types, we plot the PATS values around the pixel under consideration with corresponding direction and link the last point to the first point [6]. The resulting polygon is called the PATS polygon, and Figure 2(d) shows the calculated PATS for pixel *p* with the PATS polygons. If the road has a good contrast with its surrounding objects, the polygon usually looks like an ellipse or ∞-shape, or a circle in other cases. The compactness of PATS is defined as the compactness of the PATS polygon using Equation (4):

(4) $${\text{PATS}}_{\text{compactness}}=\frac{4\pi \cdot A}{P^{2}}$$

where *A* and *P* are the area and perimeter of the PATS polygon, respectively. It is employed to check whether the shape of the PATS polygon looks like a circle. A circle-like PATS polygon usually indicates that the tracker is no longer fit for tracking the road ahead. Note that our program will calculate the compactness of PATS at regular intervals to verify whether the PATS is still suitable for tracing a road.

## 3. Experiments and Performance Evaluation

A prototype system based on the proposed framework was developed to verify the algorithms. The hardware we used is a Dell Precision workstation T5400, with an Intel Xeon 2.50-GHz plus 2.49-GHz CPU and 3-GB RAM. It should be noted that since our system is still in the simulation stage, the program is implemented in a single thread mode. Performance should be improved if it is implemented as a multi-thread application taking full advantage of the features of multi-core processors.

### 3.1. Data collection

**Figure 3.**
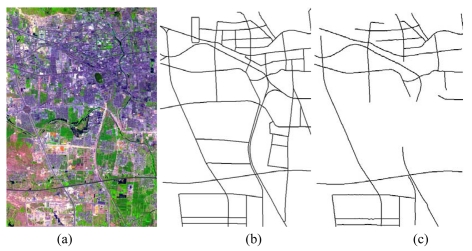
Road network extraction from a SPOT5 satellite image. (a) SPOT5 fused image with 2.5 m pixel^-1^ resolution and an image size of 3,750 pixels by 2,499 pixels; (b) the reference road network plotted; (c) the extracted vector road network by the combination strategy.

**Figure 4.**
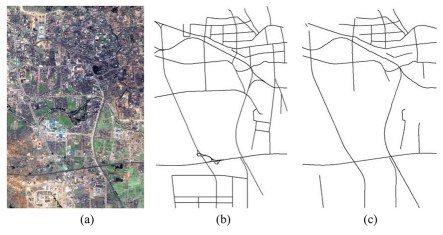
Road network extraction from an IKONOS satellite image. (a) IKONOS fused image with 1 m pixel^-1^ resolution and 9,374 pixels by 6,246 pixels image size; (b) the reference road network plotted; (c) the vector road network extracted by the combination strategy.

**Figure 5.**
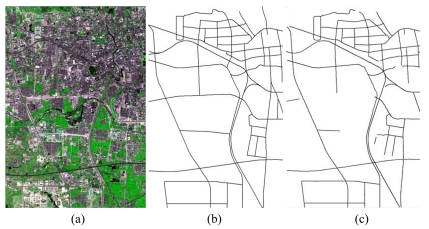
Road network extraction from a QuickBird satellite image. (a) QuickBird fused image with 0.61 m pixel^-1^ resolution and the image size is 15,368 pixels by 10,240 pixels; (b) the reference road network plotted; (c) the extracted vector road network by the combination strategy.

**Figure 6.**
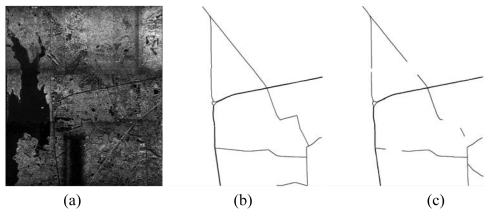
Road network extraction from an airborne SAR image. (a) Raw SAR image with 0.3 m pixel-1 resolution and 23,999 pixels by 20,172 pixels image size; (b) the reference road network plotted; (c) The extracted vector road network by PATS.

**Figure 7.**
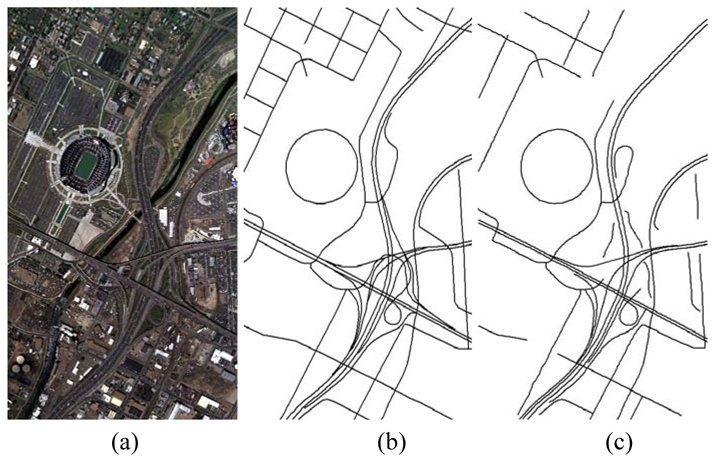
Road network extraction from a DMC airborne image. (a) DMC image with 0.2 m pixel^-1^ resolution and a 12,428 pixels by 7,780 pixels image size; (b) the reference road network plotted; (c) the extracted vector road network by combination strategy.

Various images in urban areas, suburban areas, or rural areas and images with different scales (resolutions from 2.5 m per pixel to 0.2 m per pixel) were tested to verify the capabilities of each road tracker: (1) a SPOT5 satellite image with a size of 3,750×2,499 pixels acquired in 2002 [see Figure 3(a)]. The GSD of this image is 2.5 m per pixel; (2) an IKONOS satellite image with a size of 9,374×6,246 pixels acquired in 2004 [see Figure 4(a)]. The GSD of the above image is 1 m per pixel; (3) a QuickBird satellite image with a size of 15,368×10,240 pixels acquired in 2005 [see Figure 5(a)]. The GSD of the image is 0.61 m per pixel; (4) an airborne SAR image with a size of 23,999×20,172 pixels (see Figure 6(a)). The GSD of it is 0.3 m per pixel; and (5) an aerial image acquired by a Zeiss Imaging's DMC camera with a size of 12,428×7,780 pixels [see Figure 7(a)]. The GSD of the image is 0.2 m per pixel. The images in (1), (2) and (3) are fused from their panchromatic band and multi-spectral bands by smoothing filter-based intensity modulation algorithm [36], and the geographic area for the images in (1), (2) and (3) is located at Tai'an, Shandong Province, P.R. China. Additionally, the SAR image is located at He'fei, Anhui Province, P.R. China, while the coverage area of the DMC image is within Denver, Colorado, USA.

**Figure 8.**
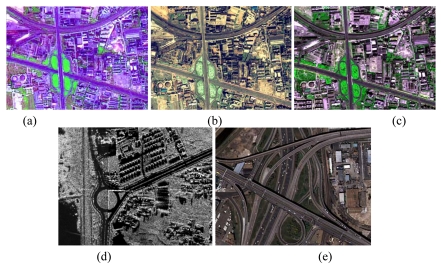
Typical roads on the images. (a) A subset of SPOT5 image in Figure 3(a); (b) a subset of IKONOS image in Figure 4(a); (c) a subset of QuickBird image in Figure 5(a); (d) a subset of SAR image in Figure 6(a); (e) a subset of DMC image in Figure 7(a).

The above five images contain the most frequent road types such as national highways and railroads, intrastate highways, ring roads, streets for local transportation, and rural roads. Furthermore, each image contains different road structures such as curves, ramps, straight roads, junctions and bridges. It also includes various road conditions such as occlusions of vehicles and shadows from trees or high buildings. The detailed characteristics of roads refer to Table 1 and Figure 8. Note that, in Table 1, the contrast means the differences between roads and other features measured by three qualitative levels: low contrast roads refer to these whose digital numbers are similar to the ones of surrounding objects while the boundaries are still discernable, mean contrast roads refer to these whose digital numbers are slightly different from the ones of surrounding objects while the boundaries are discernable and high contrast roads refer to these whose digital numbers are quite different from the ones of surrounding objects while the boundaries are discernable; the length of the roads are also measured by three qualitative levels: short one refers to that whose length is no larger than 1.0 km, mean one refers to that whose length is larger than 1.0 km but no longer than 3.0 km and long one refers to that whose length is larger than 3.0 km; the average curvature indicates the smoothness of the road measured by the levels: short one refers to that whose average curvature is no larger than 0.3, mean one refers to that whose average curvature is larger than 0.3 but no larger than 1.5 and high one refers to that whose average curvature is larger than 1.5; and the above thresholds are set based on our experience.

Based on our extensive experiments, the first threshold *I*_1_, namely the standard deviation of intensity rectangle A′B′B″A″, is set to 10.0; while the second threshold *I*_2_, namely the standard deviation of intensity rectangle A′B′B″A″, is set to 20.0. For profile matching, the coefficient *C_profile_* to calculate the width of a profile is set to 2.0, the coefficient *C_steplength_* to calculate the length of step size is set to 0.5, and the changed angle *T* for two adjacent road segments is set to 10°. For template matching, the *C_profile_* is set to 1.0, the coefficient *C_sign_* to calculate height of the template is set to 2.0, *C_steplength_* is set to 0.8, and *T* is set to 10°. For PATS algorithm, *C_profile_* is set to 1.0, *C_sign_* is set to 2.0, *C_steplength_* is set to 0.8, *T* is set to 10°, and *T*_2_ is set to 0.8. For interlaced template matching, *C_profile_* is set to 1.0, *C_sign_* is set to 0.5, *C_steplength_* is set to 0.4, *T* is set to 10°, and the width *W_sign_* of a lane marking is set to 3 pixels.

### 3.2. Evaluation criteria

Zhou *et al.* [32] proposed that the criteria used to evaluate a semi-automatic system include correctness, completeness, efficiency and accuracy. In our system, when the tracking is running after initialization, if the program detects a possible tracking problem or a tracking failure, it returns control back to the human operator. The operator supervises the road changes, diagnoses the failure reasons, deletes the incorrect parts, and restarts the tracker or inputs a new correct segment which refreshes the state model of the tracker. In this way, the correctness of tracking is guaranteed. Therefore, the completeness, efficiency and accuracy are employed to evaluate the performances of our road trackers. Particularly, the completeness is defined as the ratio of length of roads tracked by the computer with the aid of the plotter to the total length of reference data. The efficiency *C_efficiency_* is measured by time saving that can be obtained by the following formula:

(5) $$C_{\text{efficiency}}=C_{\text{completenes}}-\frac{100*T_{\text{Tracker}}}{T_{\text{Manual}}}\%$$

where *C_completeness_* is the corresponding completeness, *T_trac_*_ker_ is the time cost of the tracker including the interaction time with human operator and computation time, and *T_manual_* is time cost of fully manual plotting. Geometric accuracy is evaluated as the mean square error between the road tracker's result and the reference data. Note that the length of roads is measured in number of pixels, which is convenient to compare the performances of the same tracker on different resolutions, and the total time cost includes the computation time and interaction time with the human operator.

### 3.3. Experimental results and performance evaluation

Table 2 shows the statistical results of the performances while Figures 3, 4, 5, 6 and 7 show the raw images and the final road segments extracted from the images (1), (2), (3), (4) and (5) illustrated in Section 3.1 respectively. The profile matching method is capable of extracting the homogenous highways from the above SPOT5, IKONOS and QuickBird images with different efficiency and geometrical accuracy. But, in the SPOT5 image, even the highways are blurry due to their narrow width and low contrasts to other surrounding objects [see Figure 8(a)], which leads to frequent failures, low efficiency and large RMSE. Fortunately, this algorithm can also work on the main streets which have a sparse traffic and shadow on IKONOS image. However, profile matching is not robust compared to the other trackers, and frequent stops lead to low efficiency in spite of its low computing magnitude for per movement.

The rectangular template approach can extract the railroads and main streets besides the highways with acceptable spatial accuracy. According to the statistical results, the highest efficiency, approximately 15% of time saving, is achieved on IKONOS image. Note that the low efficiency on the SAR image is partially blamed to the noisy speckles, and mostly induced by the straightness of the roads which is in favor of manual drawing. On the other hand, the maximum completeness is achieved on the QuickBird image with 11% of time saving compared to fully manual interpretation. Unfortunately, dense traffics and shadows make this algorithm inapplicable on the DMC image. In general, this method performs better than the profile matching approach on SPOT5 image, IKONOS image and SAR image.

The PATS has the ability to extract various types of roads, but, at the same time, its performance varies considerably with images. The highest completeness occurs on the SAR image; the highest efficiency appears on the QuickBird image; and the smallest RMSE arises on the DMC image. On the other side, the lowest efficiency occurs on the SAR image, and the lowest completeness and largest RMSE appear on the SPOT5 image. As a mater of fact, the completeness and the geometrical accuracy of PATS are roughly equivalent to template matching method on the IKONOS and QuickBird images, compared to its relatively lower efficiency than the latter. Additionally, the PATS has higher completeness, lower efficiency and larger RMSE than the interlaced template matching algorithm for the DMC image test.

Interlaced template matching is proposed to extract the roads with lane markings or continuous centerlines such as highways. In fact, it not only can trace its preferred roads, but it also can extract the roads with strips of vegetations or bar-shaped features such as rails on the railroads, which is an unexpected bonus. Moreover, it is not sensitive to the dense occlusions of vehicles and tree shadows, which results in a fast tracking speed with high geometrical accuracy when it operates. In fact, its unpleasing performance on the SAR image further suggests that existing step-by-step tracking is more preponderant for smooth and curving long ribbon roads than for straight roads.

The combination strategy can extract most of the roads from the above images except the SAR image with acceptable efficiency and spatial accuracy. Particularly, the time saving reaches approximately 21% for the DMC image and approximately 33% for the QuickBird image, which can be transferred into an enhanced economic productivity in the future. Considering the potential of performance optimization of the algorithms and implementation as multiple-thread program, the result is promising.

### 3.3. Discussion

As can be seen, each road tracker has its preferred types of roads. For example, highways are favored by the interlaced template matching method, even though the surfaces of the highways may be seriously occluded by vehicles; while avenues with a few tree shadows may be traced better by the PATS on the DMC image. In fact, the combination strategy is aimed to extract each road with an optimal road tracker, which indicates its advantages from the above experiment results. However, the road trackers mentioned in this paper is not an exhaustive list. For instance, a new road tracker is needed for the fast extraction of the straight roads, and new approaches are demanded to detect and depict the various types of road junctions or crossings, which will produce a more advanced combination strategy than current one.

On the other hand, the performance of a road tracker is greatly influenced by the GSD of an image. For example, profile matching method has a higher efficiency than the interlaced template matching one in extraction of the highways from the IKONOS image, but it is not a case for the QuickBird image, because the highways on the former image has less vehicles than the latter. Unfortunately, the profile matching approach is not suitable to extract the highway from the DMC image at all, which has a very dense traffic on road surfaces [see Figure 8(e)]. A similar phenomenon occurs for the rectangular template matching algorithm. In this sense, in this paper each road tracker has its preferred GSD. Particularly, the GSD from 1 m pixel^-^ to 0.61 m pixel^-^ is favored for profile matching, from 1 m pixel^-^ to 0.3 m pixel^-^ for rectangular template matching, from 1 m pixel^-^ to 0.2 m pixel^-^ for PATS, from 1 m pixel^-^ to 0.2 m pixel^-^ for interlaced template matching method.

Additionally, complex road scenes can cause frequent tracking failures, requiring more human interactions, which significantly decreases the tracker's efficiency. As for the IKONOS image, the profile matching can track approximately 51 pixels per second, while the interlaced template matching can follow approximately 65 pixels per second. However, theoretically the speed of former should be higher than the latter considering both of the complexity of the algorithm and computing magnitude. As a mater of fact, the low efficiency is deduced by the irregular appearances of the vehicles, shadow of trees and building, junctions or crossings, etc.

Despite the consideration of decreasing the influences of the above noises for interlace template matching algorithm and PATS, large shadows of high buildings and junctions are still barriers for road tracking, especially in urban areas. In addition, currently there is no method which can more efficiently extract the straight ribbon roads with many disturbances than manual plotting, which is also one of the major reasons that decrease the overall efficiency of the combination strategy.

## 4. Conclusions

This paper presents a semi-automatic system for road tracking. The system adopts a new combination strategy to extract the road networks. Particularly, the interlaced template matching, the profile matching, the rectangular template matching, and the PATS methods are adaptively selected based on the initialization information. At the same time, a human operator is retained in the tracking process to supervise the extracted trajectory, to response to the program's prompts. Extensive experiments are performed to extract roads from aerial/satellite imagery including optical imagery and Synthetic Aperture Radar (SAR) data. The results show that the combination of multiple road trackers can more reliably and more efficiently extract most of the main roads than each individual tracker, which has significant practical applications. The current limits of our scheme are that it can't deal well with road junctions or crossings, and shadows of high buildings, and it needs a novel method to extract straight ribbon roads. Future work will also include the optimization of the algorithms to speed up the calculations.

## Figures and Tables

**Table 1.** Characteristics of the roads on the images.

**Sensors**	**Road type**	**National highway**	**Intrastate highway**	**Railroad**	**Avenue**	**Lane**
SPOT5	Contrast	low	low	low	low	-
	Average length	long	long	long	mean	-
	Average curvature	mean	mean	mean	low	-
	Noises	j	j	j	b, j	-

IKONOS	Contrast	high	mean	low	low	-
	Average length	long	long	long	mean	-
	Average curvature	mean	mean	mean	low	-
	Noises	v, j	v, j	n	v, b, c, j	-

QuickBird	Contrast	high	high	mean	mean	low
	Average length	long	long	long	mean	short
	Average curvature	mean	mean	mean	low	low
	Noises	v, j	v, j	n	v, b, c, j	v, c, j

SAR	Contrast	-	high	-	mean	-
	Average length	-	long	-	mean	-
	Average curvature	-	low	-	low	-
	Noises	-	j, s	-	j, s	-

DMC	Contrast	-	mean	mean	mean	mean
	Average length	-	long	long	short	short
	Average curvature	-	mean	mean	low	high
	Noises	-	v, c, j	j	v, b, c, j	v, b, c, j

Note that the noises include the occlusions of vehicles, shadows of buildings, occlusions of trees, road junctions or crossings, and speckles. Particularly, “v” denotes the occlusions of vehicles, “b” denotes the shadows of buildings, “c” denotes the occlusions of colonnades, “j” denotes the road junctions or crossings, “s” denotes the speckles, and “n” denotes no salient obstacles on the road surfaces.

**Table 2.** Statistics about the performance of the algorithms.

Sensors	Methods	Profile Matching	Template Matching	PATS	Interlaced Template matching	Combination	Manual
SPOT5	Length (pixels)	11045	28531	16780	-	29332	47793
	Time (seconds)	502	733	641	-	702	1444
	Completeness (%)	23.11	59.70	35.11	-	61.37	100.00
	Efficiency (%)	-11.65	8.94	-9.28	-	12.76	
	RMSE(pixels)	2.5	1.8	2.1	-	1.9	0.0
	Road Type	1,2	1,2,4	1,2,4	-	1,2,4	1,2,3,4

IKONOS	Length (pixels)	4019	55996	57332	20350	70300	108846
	Time (seconds)	782	1196	1650	312	1756	3360
	Completeness (%)	36.93	51.45	52.67	18.70	66.26	100.00
	Efficiency (%)	13.66	15.86	3.56	9.41	14.00	-
	RMSE(pixels)	1.0	1.1	1.5	0.2	1.2	0.0
	Road Type	1,2,4	1,2,3,4	1,2,3,4	1,2	1,2,3,4	1,2,3,4

QuickBird	Length (pixels)	42767	155056	16500	70579	171811	194022
	Time (seconds)	300	2077	2475	806	1695	3058
	Completeness (%)	22.04	79.92	85.05	36.38	88.56	100.00
	Efficiency (%)	12.23	11.20	4.11	10.02	33.13	-
	RMSE(pixels)	0.8	1.2	1.4	0.4	0.9	0.0
	Road Type	1,2	1,2,3,4	1,2,3,4	1,2	1,2,3,4	1,2,3,4

SAR	Length (pixels)	-	51198	98135	24498	-	110150
	Time (seconds)	-	966	1265	195	-	280
	Completeness (%)	-	46.48	89.09	22.24	-	100.00
	Efficiency (%)	-	-299.52	-366.70	-4.74	-	-
	RMSE(pixels)	-	1.5	1.3	1.4	-	0.0
	Road Type	-	2,4	2,4	2	-	2,4

DMC	Length (pixels)	-	-	123877	81241	168989	195261
	Time (seconds)	-	-	2352	820	2106	3202
	Completeness (%)	-	-	63.44	41.61	86.54	100.00
	Efficiency (%)	-	-	-10.01	16.01	20.77	-
	RMSE(pixels)	-	-	0.4	0.8	1.1	0.0
	Road Type	-	-	1.2,3,4,5	1.2,3,4	1.2,3,4,5	1.2,3,4,5

Note that road types include the ones in Table 1. Particularly, “1” denotes the national highways, “2” denotes the intrastate highways, “3” denotes the railroads, “4” denotes the avenue, “5” denotes the lane.
